# Closing the Gap: Why Percutaneous Patent Foramen Ovale and All Atrial Septal Defect Closures Belong in the NCDR IMPACT Registry

**DOI:** 10.1016/j.jscai.2026.105502

**Published:** 2026-06-30

**Authors:** Georges Ephrem

**Affiliations:** Westchester Heart and Vascular Institute, Westchester Medical Center and New York Medical College, Valhalla, New York

**Keywords:** atrial septal defect, IMPACT registry, National Cardiovascular Data Registry, patent foramen ovale, percutaneous closure

The field of structural heart disease has advanced rapidly over the past 2 decades, with transcatheter therapies transforming standards of care across age groups. Among these, patent foramen ovale (PFO) closure and atrial septal defect (ASD) closure have emerged as cornerstone interventions—PFO closure for preventing recurrent paradoxical embolic stroke^1^, ^2^, ^3^, ^4^ and ASD closure for managing the same condition as well as right-heart volume overload.^5^^,^^6^ Randomized trials have demonstrated that percutaneous PFO closure significantly reduces the risk of recurrent PFO-related stroke in carefully selected patients, particularly those with high-risk anatomical features. Similarly, percutaneous ASD closure has become the standard of care for secundum ASDs with hemodynamic significance, showing long-term improvements in right ventricular remodeling, exercise tolerance, and reduction in arrhythmia risk. However, these trials reflect controlled environments with highly experienced operators. Real-world practice is inherently more heterogeneous, and questions remain regarding outcomes in older patients, those with competing stroke etiologies, and individuals with complex septal anatomy. Complications—though infrequent—such as atrial fibrillation, device-related thrombus, and erosion require continuous surveillance.^6^

Despite robust evidence supporting their efficacy and safety, these procedures remain underrepresented in national cardiovascular registries. The only National Cardiovascular Data Registry (NCDR) to capture percutaneous ASD closure is the IMproving Pediatric and Adult Congenital Treatments (IMPACT) Registry. The NCDR IMPACT registry has already demonstrated its utility as a national benchmark for congenital and structural interventions, enabling clinicians and institutions to compare performance, identify complications, and refine procedural techniques.^7^ A mainstay of the setup of most, if not all, pediatric cardiac catheterization labs, it has not achieved a similar penetrance in adult hospitals. Accordingly, most, if not all, percutaneous ASD closures performed in an adult cardiac catheterization lab are not included in this (or any) registry. When familiar with the various parameters of percutaneous ASD closure that the IMPACT registry captures (demographics, indications, hemodynamic data, defect anatomy, procedural techniques, device details, success metrics, adverse events, and resource utilization), it becomes evident that this gap in data capture is impairing the effort aimed at enhancing patient safety, standardizing care, and enabling long-term outcome monitoring.

The situation is even more critical when it pertains to percutaneous PFO closure. The economic burden of PFO-associated strokes in the United States is substantial, exceeding $1.3 billion per year.^8^ It is estimated that as of 2023, the rate of percutaneous PFO closure had reached 29.1 per million persons,^9^ which equates to approximately 10,000 PFO closures yearly in the United States. Nevertheless, contrary to percutaneous ASD closures, there is neither a dedicated field for PFO closure in the IMPACT registry nor an obligation to log this procedure with any specific parameters in a dedicated dataset. This raises significant concerns regarding stringency of indications,^10^ quality of care, standardization of approaches, and long-term outcome monitoring in a field that was plagued by a historical controversy regarding these specific procedures with the landmark case of the 4000 closures performed by a single operator in Utah between 2002 and 2012.^11^

We therefore advocate for the inclusion of all percutaneous PFO and ASD closures in the IMPACT Registry, a national registry uniquely positioned to capture these outcomes at scale. Doing so would close a critical data gap, providing the following benefits: (1) standardized monitoring of procedural appropriateness and indication; (2) enhanced quality assurance allowing centers to benchmark outcomes, identify early complications, and drive quality improvement initiatives; (3) longitudinal follow-up systematically tracking stroke recurrence, arrhythmia, device-related thrombus, and long-term device durability; (4) device surveillance in addition to conventional postmarket monitoring ensuring patient safety and added device optimization; and (5) research opportunities as comprehensive registry data facilitate comparative effectiveness studies, identification of high-risk subgroups, and optimization of procedural strategies.

The idea of a PFO/ASD intervention registry, and specifically of including these interventions in NCDR IMPACT, has been discussed for many years. Indeed, many open questions remain in the field, including the role of closure in migraine or transient ischemic attack and the risk of post-procedure atrial fibrillation.

Understandably, there are practical considerations that make such an enterprise challenging. Unlike transcatheter aortic valve replacement, mitral and tricuspid transcatheter edge-to-edge repair and valve replacement, and left atrial appendage occlusion, there is no Centers for Medicare & Medicaid Services reimbursement-linked mandate to participate in such a registry. Most centers obviously prefer neither to hire data abstractors nor pay annual fees to participate in another registry. As for IMPACT, its current participating sites are predominantly major ACHD centers that do not reflect the sites where the majority of PFO and ASD interventions occur. Finally, the addition of all PFO and ASD closures to IMPACT could overwhelm both abstractors and the data coordinating center.

However, these challenges should not preclude progress toward a more comprehensive national data infrastructure. We have experienced firsthand the consequences of oversight absence in the field.^11^ Relying solely on commercial healthcare coverage screening or downstream judicial litigation is not a suitable solution, especially as physicians strive to regulate themselves in their own field and steer it in the desired direction, that of patient-centered care and physician-led best practice algorithms. The absence of a Centers for Medicare & Medicaid Services mandate should be remediated by professional societal advocacy for such a measure. As champions of patient and physician advocacy, institutions such as SCAI should lead the effort in advocating for and implementing such a measure. The costs incurred would be totally compensated by the protection of the patients’ well-being and the physicians’ reputation and integrity that occurrences such as the Utah case can disastrously jeopardize, especially as the spectrum of indications for these procedures may expand.

Achieving this goal can be approached in a feasible manner. Rather than immediate universal implementation, a phased strategy could be considered starting with programs where a substantial proportion of these procedures are currently performed and eventually including the totality of sites. Such a gradual approach would provide time for strategic implementation of the necessary steps, including using staff already assigned to other NCDR registries to cover such a task and leveraging artificial intelligence for data extraction and submission without incurring immediate financial impact. Such an approach would leverage an established registry infrastructure while minimizing fragmentation of data collection efforts.

Inclusion of PFO and ASD closure within a national registry also aligns with the broader mission of the NCDR to capture the evolving spectrum of cardiovascular care. Beyond research applications, registry participation could provide meaningful value to centers through benchmarking, quality improvement initiatives, postmarket surveillance, and real-world evidence generation that may ultimately inform clinical guidelines, payer policy, and appropriate-use frameworks.

Some may argue that the relatively low complication rates associated with PFO and ASD closure do not justify large-scale registry inclusion. However, this perspective underestimates both procedural volume and clinical complexity, particularly as indications expand and procedures increasingly occur across diverse practice settings. Moreover, leveraging an existing infrastructure such as IMPACT may prove substantially more efficient than developing fragmented or duplicative data silos.

In conclusion, percutaneous PFO and ASD closures are safe, effective, and increasingly utilized interventions. Their inclusion in the NCDR IMPACT registry is not merely administrative—it is a necessary evolution in interventional data infrastructure. Systematic data collection would standardize outcome reporting, enhance patient safety, and generate real-world evidence that informs best practices and policy decisions. As structural heart interventions continue to advance, our data systems must evolve accordingly. The question is no longer whether these procedures warrant inclusion, but why this integration has not yet been prioritized and how best to implement it in a practical, sustainable, and inclusive manner.

## Declaration of competing interest

The author declared no potential conflicts of interest with respect to the research, authorship, and/or publication of this article.
